# New York State Veterinary Medical Society

**Published:** 1897-11

**Authors:** Claude D. Morris

**Affiliations:** Secretary


Secretary.

### NEW YORK STATE VETERINARY MEDICAL SOCIETY.

THE eighth annual meeting convened in the assembly room of the Vanderbilt, Syracuse, September 14th, at 11 A.M. As neither the President nor Vice-President was present, Dr. Roscoe R. Bell, of Brooklyn, N. Y., was chosen President *pro tem.*, who offered a few remarks in expression of his appreciation of the courtesy the members had shown in asking him to preside over the deliberations of the Society. On roll-call, the following were present: Drs. E. F. Bettinger, J. A. Bell, R. R. Bell, W. L. Baker, H. E. Clark, J. M. Currie, Charles Cowie, W. S. Corlis, E. M. Casey, G. C. Dean, H. D. Gill, M. Henderson, Wilson Huff, V. L. James, F. L. Kilborne, G. C. Kelser, W. H. Kelly, James Law, V. A. Moore, F. Morrow, C. D. Morris, J. A. McCrank, Arthur O'Shea, Thomas O'Dea, R. Perkins, M. N. Poucher, W. H. Pendry, J. A. Pendergast, J. L. Ronan, W. B. Switzerm, A. G. Wicks. The record of the previous meeting was read and submitted for corrections; there being no errors, it was accepted and ordered spread on the minutes.

The Secretary then gave his annual report, which was an exhibit of the work of the office, the finances of the Society, the growth of the profession in the State as it relates to both physical and literary achievement.

The Board of Censors reported favorably on the following applications for membership: Charles F. Ebner, Syracuse; E. F. Bettinger, Chittenango; E. M. Casey, Oxford; H. E. Clark, Newburgh; F. L. Kilborne, Kelloggsville, and George Eighmy, North Chatham; and recommended the following on the application of John P. O'Leary, vouched for by Drs. Hinkley and Willyoung: "Inasmuch as this application comes down to us without action from a previous Board of Censors, and that the facts that led to their laying it on the table are not before us, we recommend that action be delayed for further investigation." The Board further recommended that the Society accept the resignation of J. H. Ferster, of New York City, and to hold over the resignation of Herbert F. Neher, of New York City, on the ground of his indebtedness to the Society. Report of the Board of Censors was accepted. A motion prevailed that the Secretary cast an affirmative ballot for the applicants favorably reported.

A motion to adjourn for lunch was carried.

*Afternoon Session.* Meeting convened at 1.30 o'clock. The newly elected members were introduced to the sitting members of the Society with a few well-chosen remarks by Dr. R. R. Bell, the presiding officer, assuring them of the fraternal feeling on the part of the Society in their behalf, and that in their new social relations to the profession much would be expected of them.

The report of the various committees was in order on the programme. The Executive Committee had nothing to report.

The By-laws Committee reported on the motion to amend Article VIII., Chapter I., in relation to annual dues, so as to read, "the dues shall be three dollars per annum, payable in advance." The amendment to raise the annual dues from two dollars to three dollars per annum was strongly opposed by Drs. Law, Gill, and Switzer. The question being called, the vote resulted in favor of the negative, twenty to five.

The second amendment to Article VIII., Chapter I., relating to the initiation fee and annual dues accompanying the application of future candidates, was carried by unanimous consent. If such application is accepted, such fees and dues to be appropriated to the use of the Society; if rejected, the money to be refunded.

The report by the County Secretaries was both interesting and instructive. The reports show plainly that the Society has inaugurated a movement in behalf of sanitary work which is producing excellent results. Several local boards of health now employ a veterinarian, whose duty it is to inspect the meat- and milk-supply of his neighlorhood or district.

The Committee on Legislation, through its chairman, Dr. Kelly, rendered a full report of work done during the last session of the Legislature. This report was accepted. A supplementary report of this work was submitted by Professor Law, a member of the Legislative Committee, which report dealt with matters pertaining to the Public Health Department of the State, and its relation to the Tuberculosis Commission, with a suggestion that, inasmuch as the Department of Health and Agriculture could not agree on matters supporting the Tuberculosis Commission, that a new commission be formed, composed of three men, namely, the Secretary of the State Board of Health, the Commissioner of Agriculture, and the Director of the New York State Veterinary College. In support of this compromise it was shown by Drs. Law, Kelly, and Morris that such a commission would represent all interests and harmonize whatever feeling may exist in these two departments toward each other in support of a great sanitary work, and that in the end the public would reap the greatest benefit. This feature of Professor Law's report brought forth a vigorous protest from Drs. Gill and Bell, each representing respectively the Veterinary schools of New York City. Their argument was that such a measure ignored the pioneer veterinary colleges of the State, and that the tendency at the State College (through the Director) was to centralize the function of the veterinary profession throughout the State in the interests of the State College. After some further discussion of the matter the question was ordered to be laid on the table.

Reading of professional papers being in order, Professor Law gave a very instructive paper on "How to Prepare Contagious Products for Shipment to the Pathological Laboratory." The information this paper imparted on that subject outlined the essentials necessary to successfully prepare and ship to the laboratory any pathological specimen. To the painstaking and inquiring veterinarian this paper is of great value.

Dr. V. A. Moore, of the State College, read a paper entitled, "Poisoning by Carbonate of Soda, a Cause of Death Among Swill-fed Hogs," and also with the aid of charts gave "The Distinction between Hog-cholera and Swine-plague: the Post-mortem Appearances as Usually Observed in these Diseases." Dr. Moore's paper and remarks were very instructive. Few men are able to distinguish between these two diseases, but we feel that every one who heard Dr. Moore on this subject added to his store of professional knowledge a rich supply of material touching an important question of a disease which is quite obscure and which is constantly working eastward, and this year to a greater extent than ever. The paper brought forward many questions of inquiry, and was discussed all round the room.

A motion prevailed to hold an evening session. Adjourned for dinner to convene again at 7.30 P.M.

*Evening Session.* The President appointed the following-named gentlemen a Committee on Resolutions: Drs. Gill (Chairman), Kelly, O'Shea, Pendry, and Morris.

Dr. W. L. Baker read a paper on the "Uses of Cold in the Treatment of Laminitis." The merits of such treatment were discussed freely. The meeting adjourned.

*September 15th*: Meeting called to order at 11 o'clock. Morning session opened by the reading of a paper by Professor Roscoe R. Bell, of the American Veterinary College; subject, "Infectious Catarrhal Fever of Horses." Dr. Bell's paper covered every phase of this disease. The etiology, pathology, and treatment of this disease were handled in a clear, concise manner. The paper was very instructive, eliciting much discussion.

"Caudal Surgery; its Objects and Results," were clearly defined from the pen of Dr. W. L. Williams, of the State College. Dr. Williams being unable to attend the meeting, Professor Law read the paper.

Dr. Charles Cowie gave an instructive paper on "Thoroughpin and its Treatment." His method is to draw off the bursal fluid and then fire the lining membrane of the sac, a mode of treatment among some veterinarians in France thirty-five years ago. Dr. Bell, of Watertown, treats thoroughpin by the same method, and both he and Dr. Cowie report excellent success from this kind of treatment. The opinion of many in the room was that Dr. Cowie had unlocked an old treasure-box and exhibited something new.

The next paper on the programme was "Veterinarians as Jurors," by Dr. Morris, who called attention to the principle that as citizens and professional members of society we have no right or just excuse to refuse to bear our just proportion of the obligations we owe to the public, which coexist with the function of citizenship; that shirking is skulking from a common duty, and gives semblance to the impression that the men of the profession refuse to uphold one of the fundamental laws of the land, thus showing their disrespect for a time-honored institution, which has become a bulwark in the fabric of civilized nations. A motion prevailed to adjourn for lunch.

The meeting convened at 2 P.M. Committee on Resolutions reported that nothing had been presented. Dr. Huff, of Rome, presented a communication relative to sanitary work performed by their local Board of Health among the dairies suplying that city with milk; also read newspaper extracts in criticism of that work. A motion prevailed directing the Secretary to communicate with the health authorities of the city of Rome, tendering them an expression of approval for the excellent sanitary work they were carrying out in behalf of the consumers of dairy-products and animal flesh.

Nomination and election of officers being next in order, the following-named gentlemen were elected for two years: W. L. Baker, of Cortland, President; Roscoe R. Bell, of Brooklyn, Vice-President; and C. D. Morris, of Pawling, Secretary-Treasurer. The censors elected were Professor James Law, of the State College, Ithaca; W. H. Kelly, Albany; M. M. Poucher, Oswego; Wilson Huff, Rome; H. D. Gill, New York City.

A motion to consider a place for holding the next annual meeting was carried; and, after a brief discussion on the question, by balloting New York City was chosen by a majority of one.

Some of the papers read at the meeting will appear in print.

A motion to adjourn was carried.

CLAUDE D. MORRIS,
Secretary.

